# Frequency of the T307A, N680S, and -29G>A single-nucleotide polymorphisms in the follicle-stimulating hormone receptor in Mexican subjects of Hispanic ancestry

**DOI:** 10.1186/s12958-018-0420-4

**Published:** 2018-10-19

**Authors:** Gabriela García-Jiménez, Teresa Zariñán, Rocío Rodríguez-Valentín, Nancy R. Mejía-Domínguez, Rubén Gutiérrez-Sagal, Georgina Hernández-Montes, Armando Tovar, Fabián Arechavaleta-Velasco, Patricia Canto, Julio Granados, Hortensia Moreno-Macias, Teresa Tusié-Luna, Antonio Pellicer, Alfredo Ulloa-Aguirre

**Affiliations:** 1 Instituto Valenciano de Infertilidad (IVINSEMER), Thiers 96, Col. Nueva Anzurez, CP 11590 Mexico City, Mexico; 2 Red de Apoyo a la Investigación (RAI), Coordinación de la Investigación Científica, Universidad Nacional Autónoma de México (UNAM)-Instituto Nacional de Ciencias Médicas y Nutrición SZ (INCMNSZ), Calle Vasco de Quiroga 15, Tlalpan, 14000 Ciudad de Mexico, Mexico; 3 Centro de Investigación en Salud Poblacional, Instituto Nacional de Salud Pública, Av. Universidad 655, CP 62100 Cuernavaca, Mor Mexico; 4 Department of Physiology of Nutrition, INCMNSZ, Mexico City, Mexico; 5 Department of Transplantation, INCMNSZ, Mexico City, Mexico; 6 Research Unit in Reproductive Medicine, UMAE Hospital de Ginecoobstetricia “Luis Castelazo Ayala”, Río de la Magdalena 289, Tizapán San Ángel, Mexico City, 01090 Mexico; 7 Facultad de Medicina, UNAM, Ciudad Universitaria, Mexico City, Mexico; 8 Universidad Autónoma Metropolitana-Iztapalapa, Av. San Rafael Atlixco 186, Leyes de Reforma 1ra. Secc., Mexico City, 09340 Mexico; 9 Unidad de Biología Molecular y Medicina Genómica, UNAM-INCMNSZ, Mexico City, Mexico

**Keywords:** Follicle stimulating hormone, Oocyte donation, Ovulation induction, Assisted reproductive techniques, Infertility, Female infertility, FSHR gene SNPs

## Abstract

**Background:**

*FSHR* SNPs may influence the ovarian sensitivity to endogenous and exogenous FSH stimulation. Given the paucity of data on the *FSHR* c.919A **>** G, c.2039A > G and − 29G **>** A SNPs in Hispanic population, we here analyzed their frequency distribution in Mexican mestizo women.

**Methods:**

Samples from 224 Mexican mestizo women enrolled in an IVF program as well as a genotype database from 8182 Mexican mestizo subjects, were analyzed for *FSHR* SNPs at positions c.919, c.2039 and − 29G > A. Association between the genetic variants and reproductive outcomes was assessed.

**Results:**

The c.919 and c.2039 SNPs were in strong linkage disequilibrium and their corresponding genotype frequencies in the IVF group were: AA 46.8%, AG 44.2%, and GG 8.9%, and AA 41.9%, AG 48.2% and GG 9.8%, respectively. For the -29G > A SNP, genotype frequencies were 27% (GG), 50% (GA) and 23% (AA). In normal oocyte donors with the c.2039 GG genotype, the number of oocytes recovered after ovarian stimulation (COS) were significantly (*p* < 0.01) lower than in those bearing other genotypes in this or the -29G **>** A SNP. Analysis of the large scale database revealed that both allelic and genotype frequencies for the three SNPs were very similar to those detected in the IVF cohort (*p* ≥ 0.38) and that female carriers of the c.2039 G allele tended to present lower number of pregnancies than women bearing the AA genotype; this trend was stronger when women with more Native American ancestry was separately analyzed (OR = 2.0, C.I. 95% 1.03–3.90, *p* = 0.04). There were no differences or trends in the number of pregnancies among the different genotypes of the -29G > A SNP.

**Conclusions:**

The frequency of the GG/GG combination genotype for the c.919 and c.2039 SNPs in Mexican hispanics is among the lowest reported. The GG genotype is associated with decreased number of oocytes recovered in response to COS as well as to lower pregnancy rates in Hispanic women from the general population. The absence of any effect of the -29AA genotype on the response to COS, indicates that there is no need to perform this particular genotype testing in Hispanic women with the purpose of providing an individually-tailored COS protocol.

**Electronic supplementary material:**

The online version of this article (10.1186/s12958-018-0420-4) contains supplementary material, which is available to authorized users.

## Background

Follicle-stimulating hormone (FSH), one of the gonadotrophins synthesized by the pituitary gland, plays a pivotal role in reproduction. This gonadotrophin binds its cognate receptor, the follicle-stimulating hormone receptor (FSHR), in the granulosa cells of the ovarian follicles and the Sertoli cells lining the seminiferous tubules of the testes, to regulate an array of biological effects associated with reproductive competence. In the ovary, FSH stimulates follicle growth and maturation, as well as the synthesis of estrogens, whereas in the testes it supports spermatogenesis [1, 2].

Of the nearly 2000 single nucleotide polymorphisms (SNPs) of the *FSHR*, five are located in exon 10 [3]. Four of these SNPs are non-synonymous and lead to amino acid substitution, resulting in the T307A, R524S, A665T, and N680S FSHR protein variants [4, 5]. The most common and best studied SNPs of this receptor are c.919A > G (rs6165) and c.2039A > G (rs6166), which are inherited in strong linkage disequilibrium [at least in Caucasians and Asians and less in Africans [6]] and whose most common FSHR variants, T307/N680 and A307/S680, are almost equally distributed among Europeans [3, 6–9].

A number of studies indicate that FSHR function is influenced by the p.T307A and p.N680S polymorphisms. In particular, the p.N680S SNP has received special attention because of its association with variations in the sensitivity of the FSHR to its cognate agonist and the ovarian response to FSH stimulation as disclosed by in vitro [10, 11] and in vivo studies [reviewed in [4, 7, 8, 12]]. More specifically, young women with the GG genotype tend to present lower ovarian sensitivity to endogenous FSH, which apparently leads to higher pituitary FSH secretion and longer duration of the menstrual cycle compared to women with the AA genotype [13]. This altered FSHR sensitivity to agonist, frequently makes necessary personalization of the controlled ovarian stimulation (COS) protocol, usually by administering higher FSH doses to overcome the decreased ovarian response provoked by the N → S substitution at position 680 of the FSHR [8, 14–17]. Moreover, this particular SNP has been proposed as a predictive biomarker for determining the optimal FSH dose to be used in COS protocols [8, 9, 13, 14, 18]. Apparently, the negative effect of the S680 variant on the FSHR response to agonist decreases with age and fertility status [19–21]. The N680S SNP also has been linked with other abnormalities [22–26], including lower testicular volume in selected North European populations bearing the S680S genotype variant, particularly when it coexists with the *FSHB* -211G > A SNP [27].

The less studied −29G > A polymorphism, located in the core promoter region of the *FSHR* (rs1394205), has been associated with reduced transcriptional activity of the receptor gene in women with the -29AA genotype, as well as to primary or secondary amenorrhea and poor response to exogenous FSH in selected populations [28–31]. The major A allele frequency of this SNP ranges from 50 to 70% in East Asia and Europe [6].

Data on these *FSHR* SNPs in Hispano-American population are rather scarce, and the only available data comes from the HapMap and the 1000 Genomes Project database obtained in a small cohort of Mexican-American subjects with Mexican origen residing in Los Angeles, CA, USA [32]. In this particular population and according to the HapMap and the 1000 Genomes Project databases, the allele and genotype frequencies of the c.2039 GG SNP variant ranges from 33 to 34.0% and from 6.0 to 7.8%, respectively, whereas for the -29G > A SNP, these data bases indicate frequencies of 26% to 33% for the AA genotype and 49% to 55% for the A allele (http://grch37.ensembl.org), respectively.

The primary objective of the present study was to analyze the frequency distribution of these common *FSHR* SNPs in Mexican subjects of Hispanic origin, based on data obtained in larger populations than those previously included in reported databases [32]. For this purpose, we analyzed samples and data from three distinctly different groups of subjects in order to obtain the most accurate prevalence values and also to examine the influence of ancestry on the frequency estimates observed in Mexican mestizos. As secondary objectives, we examined the potential associations between the c.2039A > G and -29G **>** A SNPs genotypes with various outcomes of the COS protocol applied to women belonging to one of the study groups, as well as with some reproductive parameters extracted from the large database used as a reference.

## Subjects and methods

Three different groups of Mexican subjects with Hispanic ancestry were included in the study: a cohort of normal and infertile Mexican mestizo women attending a private assisted reproduction clinic in Mexico City (IVF group); a group of 100 normal Mayan mestizo women with low Mayan-Spaniard miscenegation; and a population belonging to a large database of Mexican mestizo subjects in whom data on allelic and genotype frequencies of these SNPs were available.

### IVF group

The first study group (IVF group) was conformed by a cohort of 224 Mexican mestizo women [80 normal oocyte donors aged 18 to 29 years (median, 24 years) and 144 infertile patients aged 22–43 years (median, 35 years)] who attended the Instituto Valenciano de Infertilidad-Mexico (IVI) and accepted to participate in the study. All participants in this group were unrelated and of self-reported Mexican mestizo ancestry (at least 3 generations), and both the treating physician and the volunteer were blind to the genotyping results until the end of the study. Women in the donor group were eligible whenever they met the criteria established by the IVI for oocyte donors, including normal karyotype, age between 18 and 30 years, and normal follicular reserve as assessed by intravaginal ultrasound. Inclusion criteria for the infertile group included: *a.* Presence of both ovaries without morphological abnormalities, except when the diagnosis of polycystic ovary syndrome (PCOS) was established [according to the Rotterdam criteria [33]]; *b.* Both ovaries adequately visible by intravaginal ultrasound; *c.* Absence of any endocrinological disease or obesity, except hypothyroidism under treatment or PCOS; and *d.* Any cause of infertility, including tubal factor, endometriosis, male factor, mixed (female/male) factor, and unknown cause.

For COS, women were prepared with an oral contraceptive (OC) (ethynilestradiol 30 μg plus drospirenone 3 mg; Bayer Schering Pharma, Berlin, Germany) for 10–21 days in the cycle preceding the COS cycle and then treated with gonadotrophin-releasing hormone antagonist (GnRHa) and menotropins (highly purified LH/FSH, 1:1; Merapur®, Ferring, Mexico) with or without recombinant human FSH (recFSH; Gonal F®, Merck-Serono, Mexico) as add-on treatment, or with GnRHa and recFSH. All women presented withdrawal bleeding after discontinuation of the OC. Ovarian stimulation was started 5 days after discontinuation of the OC with 150 to 225 IU menotropins, 75 IU menotropins plus 150 IU recFSH, or 150 IU recFSH after establishing ovarian and uterine quiescence by intravaginal ultrasound. Gonadotrophins were administered in a step-up fashion, adjusting the dose every 3 to 4 days depending on the ovarian response and the criteria of the treating physician, following stimulation protocols well established by the IVI. When the mean diameter of the leading follicle reached 14 mm as disclosed by intravaginal ultrasound, daily s.c. injections of 0.25 mg Cetrorelix (Cetrotide; Merck Serono S.A. Mexico) were added to gonadotrophin treatment until one or more follicles reached a mean diameter of 18 mm, time when 250 μg s.c. recombinant human chorionic gonadotrophin (hCG) (Ovidrel®, Merck Serono S.A., Mexico) was administered. Transvaginal ultrasound-guided oocyte retrieval was performed 36 h after hCG injection.

As secondary objective for the IVF study group, the response to COS was recorded and analyzed for differences among women with distinct N680S and -29G/A SNPs. To accomplish this, data containing total FSH and LH administered, serum estradiol [measured by a commercial chemiluminescence immunoassay (Beckman Coulter Life Sciences, Indianapolis, IN, USA)], number of oocytes recovered, and days of stimulus required to reach a mean follicle diameter of 18 mm, were collected from donors and patients who completed the stimulation cycle until oocyte retrieval. Women who did not complete the COS cycle for any reason (either voluntarily or because of risk of hyperstimulation, poor response in terms of number of growing follicles, low serum E2 levels, and/or asynchrony in follicular growth), as well as patients with the diagnosis of PCOS (a condition that may influence on the ovarian response to COS) were excluded from the secondary analysis. To explore for differences in response among women with different N680S FSHR variants and -29G/A SNP genotypes and to minimize bias in the analysis of the results, we first examined separately in the group of donors and infertile patients who completed the stimulation cycle for homogeneity in the distribution of gonadotrophin treatment, age, and diagnosis (in the infertile group) among the three genotypes of each SNP, and thereafter analyzed within each group the effect of the genotype on the secondary outcomes controlling for gonadotrophin treatment [grouped as follows: *a.* recFSH treatment; *b.* LH/FSH (menotropins) treatment; and *c*. Mixed (menotropins *plus* recFSH) gonadotrophin treatment] and the other parameters.

### Mayan mestizo women

The second population group studied was conformed by 100 normal Mayan mestizo women, aged 16 to 37 years (median, 20 years), resulting from the admixture between Mayan and Spaniard population with at least one Mayan surname, and whose DNA was analyzed to determine the frequency of the N680S FSHR variant and the impact of the Spaniard ancestry on the presence of this particular *FSHR* SNP in the Mexican mestizo population. Other *FSHR* SNPs were not analyzed in this group due to insufficient DNA sample available. Data on this particular population has been previously reported [34].

### Mexican mestizo subjects from a large database

To compare the allelic and genotype frequency of the *FSHR* SNPs found in the above described groups with those from an open Mexican population, a third group of data (SIGMA cohort) from a large database genotyped using the Illumina OMNI 2.5 array was analyzed. This reference sample was conformed by 8182 Mexican mestizo subjects participants in the Slim Initiative in Genomic Medicine from the Americas (SIGMA) Type 2 Diabetes Consortium [35] [3515 (43%) male and 4667 (57%) female; 4366 (53%) non-diabetic and 3848 (47%) subjects with type 2 diabetes (T2D), all exhibiting Native American and European ancestry as determined by Principal Components Analysis [36]]. Details on the selection criteria, quality control procedure, and estimation of Native American and European ancestry proportions have been reported elsewhere [35]. In our secondary analysis of this reference database, information related with reproductive events such as age at menarche and menopause, and number of pregnancies in a subset of 520 women (aged 34 to 89 years, median 52 years) were extracted from this database (UIDS cohort; [35]) and analyzed for potential associations with the *FSHR* SNPs studied.

### FSHR genotyping in samples from the IVF group and the Mayan women

Total DNA was extracted from peripheral blood lymphocytes employing the QIAamp DNA Blood Mini kit (Qiagen Inc., Valencia, CA, USA) and purified using the Wizard Genomic DNA Purification Kit (Promega, Madison, Wisconsin, USA) following the manufacturers’ instructions. Analysis of the *FSHR* SNP at position 2039 (N680S) was carried out using a predesigned TaqMan allelic discrimination assay for the StepOne plus system (Applied Biosystems, Inc., Foster City, CA, USA). The results from the TaqMan assay were verified in all samples by PCR-restriction fragment length polymorphism (RFLP) as previously described [37]. In this IVF group, SNPs at positions 919 (T307A) and -29 also were analyzed by PCR-RFLP following the methods and oligonucleotide primers reported by Sudo et al. [37] and Achrekar et al. [28], respectively. For the 3 polymorphisms, the specificity and validity of the TaqMan and RFLP procedures were confirmed in 10% of the PCR products obtained (randomly selected from all samples processed) by direct sequencing. The procedure employed for determining the SNP genotypes in the SIGMA cohort has been described in detail elsewhere [35].

### Statistical analysis

#### Data from the IVF group and Mayan mestizo women

Differences in allelic and genotype frequencies between women included in the IVF group (donors and infertile patients) and Mayan mestizo women were analyzed using the chi-squared test, with Yates’ correction for the case of the allelic frequency of the -29G > A SNP.

For the analysis of the secondary objectives in the IVF group and given that the study was not originally designed with the power to evaluate the above described associations among different COS outcomes and genotypes, we first determined whether gonadotrophin treatment and diagnosis (in the case of the group of patients) were homogeneously distributed among the different genotypes and then analyzed for the existence of significant differences in secondary outcomes. To test for homogeneity of gonadotrophin treatment, age, and diagnosis vs genotype, the chi-squared test was employed. Differences in secondary endpoints (dose of gonadotrophins administered, serum E2 levels, days of COS, and number of oocytes retrieved) among genotypes in the donors and infertile patients were then analyzed by a generalized linear mixed model (GLMM), considering genotype as the fixed factor and hormonal treatment, age and diagnosis as random factors [38]. The GLMM test was chosen considering that the study was not originally designed to analyze for differences in secondary outcomes among genotypes and that this test allowed to control simultaneously for age, treatment, and diagnosis (in the case of infertile patients). A GLMM with gamma error was employed to seek for differences in LH doses administered and serum E2 levels, whereas a GLMM with Poison’s error was used to calculate for differences in the number of oocytes recovered and days of COS. The Tukey’s test was employed as post-hoc test for the effect of the genotype on oocyte number in donors. Although the number of secondary outcomes compared was relatively small, correction for multiple testing was anyway performed employing the Bonferroni’s correction procedure [39].

#### Analysis of data from the SIGMA cohort

Pairedwise proportions test was employed to compare genotype frequencies between the IVF group and SIGMA subjects. Logistic regression models adjusted for ethnicity were employed to explore potential associations between *FSHR* genotypes and some reproductive outcomes such as age at menarche and menopause, and number of pregnancies in the UIDS cohort (see above). When the latter outcome was analyzed, the age was added as covariate in the model.

Linkage disequilibrium in the *FSHR* SNPs variants detected in the IVF group and SIGMA cohort was determined using the Haploview version 4.1 [40], in which *D’* = D/Dmax (where D is the deviation of the observed from the expected) and *r*^*2*^ is the correlation coefficient between pairs of loci. The maximum values of *D’* and *r*^*2*^ are 1.000, which indicate complete linkage disequilibrium or pairwise correlation between the loci, respectively.

Since the Mayan population studied did not followed Hardy-Weinberg equilibrium for the c2039A > G SNP, and considering that women in the Mexican culture preserve both parents’ surnames and that even if married they inherit the parental surnames to their descendants, we further compared the surnames of the Mayan population studied and tested for population equilibrium following the method described by Lasker [41].

## Results

### Frequency of the T307A, N680S and -29A/G variants in the IVF cohort

**Fig. 1:**
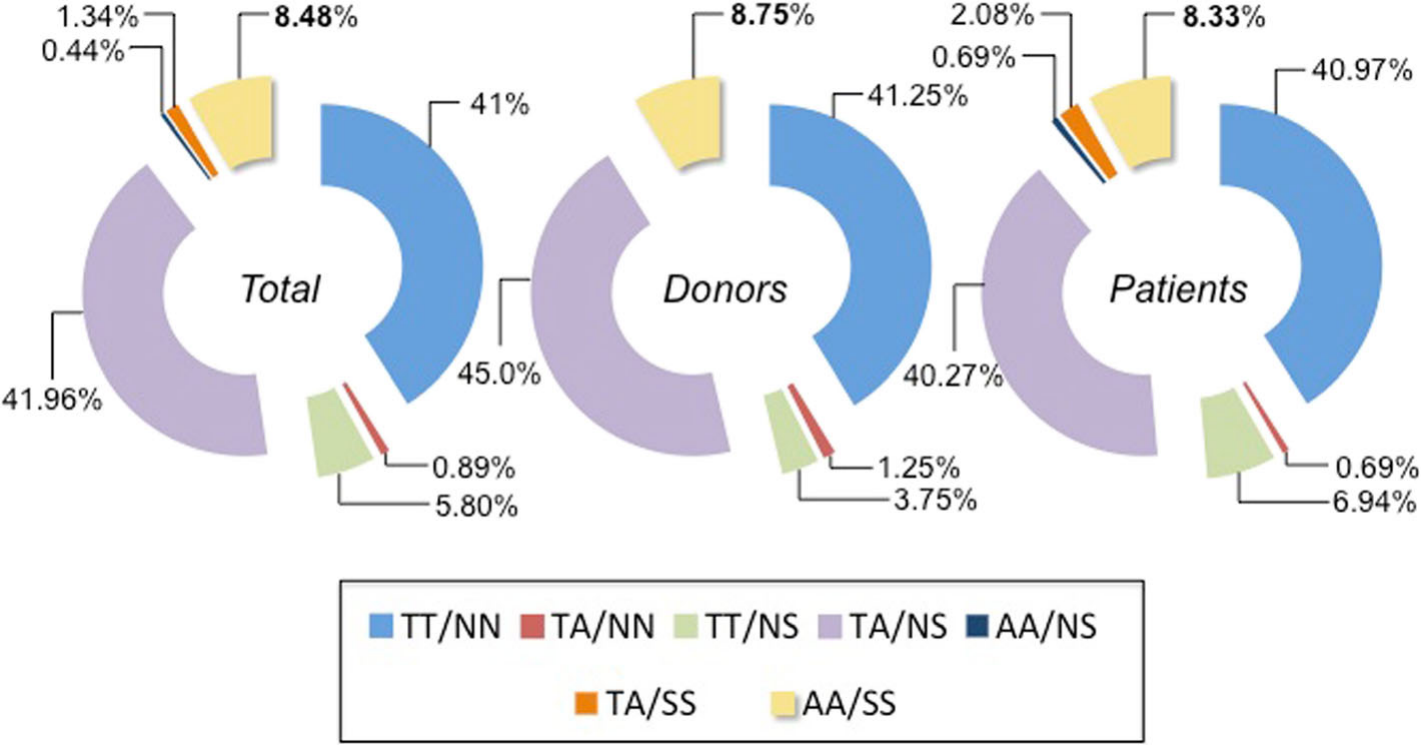
Frequency distribution of the haplotypes at positions 307 and 680 of the FSHR protein. The homozygous TT/NN and heterozygous TA/NS haplotypes were the two most frequently observed combinations in Mexican mestizo women. The minor homozygous haplotype (AA/SS) was detected in only 8–9% of all women

The allele and genotype frequency for the T307A and N680S SNPs in the IVF group with 224 women (donors and infertile women) studied are presented in Table 1. As shown, the allelic frequencies for the SNPs at positions 307 and 680 of the FSHR in the donor and patient groups were virtually identical. Overall, the allelic frequencies for the A and G alleles at position c.919 (p.T307A) were 69% and 31%, whereas for those at position c.2039 (p.N680S) were 66% and 34%, respectively. Genotype frequencies at position c.2039 were also very similar between the two groups of women: the GG genotype (p.S680 in both alleles) which appears to influence the ovarian response to COS [9] was 8.7% in the donors while in the infertile women it was 10.4% (*p* = 0.792), yielding a mean frequency of 9.8% in the whole population studied. The frequency distribution of homozygous and heterozygous women for the T307A and N680S SNPs is shown in Fig. 1. As shown in this figure, the frequency distributions of the TT/NN and AA/SS haplotypes in donors and infertile patients were virtually identical [41.25% vs 40.97% (TT/NN), and 8.75% vs 8.33% (AA/SS) in donors and patients, respectively]. Nearly 42% of all women (45% of normal donors and 40% of patients) were heterozygous (TA/NS) for both alleles and the frequency was low for heterozygosity in only one allele (0.44% to 5.8%), with the lowest being the AA/NS haplotype combination, followed by TA/NN, TA/SS, and TT/NS. In this study group, the distribution of the SNPs (including the -29G > A SNP, see below) followed Hardy-Weinberg equilibrium. Analysis of the association between the rs6165 and rs6166 SNPs in the IVF group revealed a strong linkage disequilibrium, with *D’* = 0.997 (0.970 and 0.918 in donors and patients, respectively) and *r*^*2*^ = 0.818 (0.889 and 0.781 in donors and patients, respectively), and minor allele frequencies (MAG) of 0.309 (rs6165, c.919A > G) and 0.339 (rs6166, 2039A > G) for the whole group.

**Table 1:** Allele and genotype frequencies for the single nucleotide polimorphism (SNP) c.919A > G and c.2039A > G of the *FSHR* in the population of normal oocyte donors and infertile Mexican mestizo women

Group	SNP	Allele frequency %	Genotype frequency %
Donors (*n* = 80)	c.919A > G (p.T307A)	A (T) 68.1^&^	AA (TT) 45.0*
		G (A) 31.8	AG (TA) 46.2
			GG (AA) 8.7
	c.2039A > G (p.N680S)	A (N) 66.8^&&^	AA (NN) 42.5**
		G (S) 33.1	AG (NS) 48.7
			GG (SS) 8.7
Infertile women (*n* = 144)	c.919A > G (p.T307A)	A (T) 69.4	AA (TT) 47.9
		G (A) 30.5	AG (TA) 43.0
			GG (AA) 9.0
	c.2039A > G (p.N680S)	A (N) 65.6	AA (NN) 41.6
		G (S) 34.3	AG (NS) 48.0
			GG (SS) 10.4
TOTAL (*n* = 224)	c.919A > G (p.T307A)	A (T) 68.9	AA (TT) 46.8
		G (A)31.0	AG (TA) 44.2
			GG (AA) 8.9
	c.2039A > G (p.N680S)	A (N) 66.0	AA (NN) 41.9
		G (S) 34.0	AG (NS) 48.2
			GG (SS) 9.8

^&^*P* = 0.930 and ^&&^*P* = 0.987 vs infertile women for the A and G alleles at positions c.919 and c.2039, respectively

*P = 0.897 and **P = 0.922 vs infertile women for the AA, AG, and GG genotypes at positions c.919 and c.2039, respectively

**Fig. 2:**
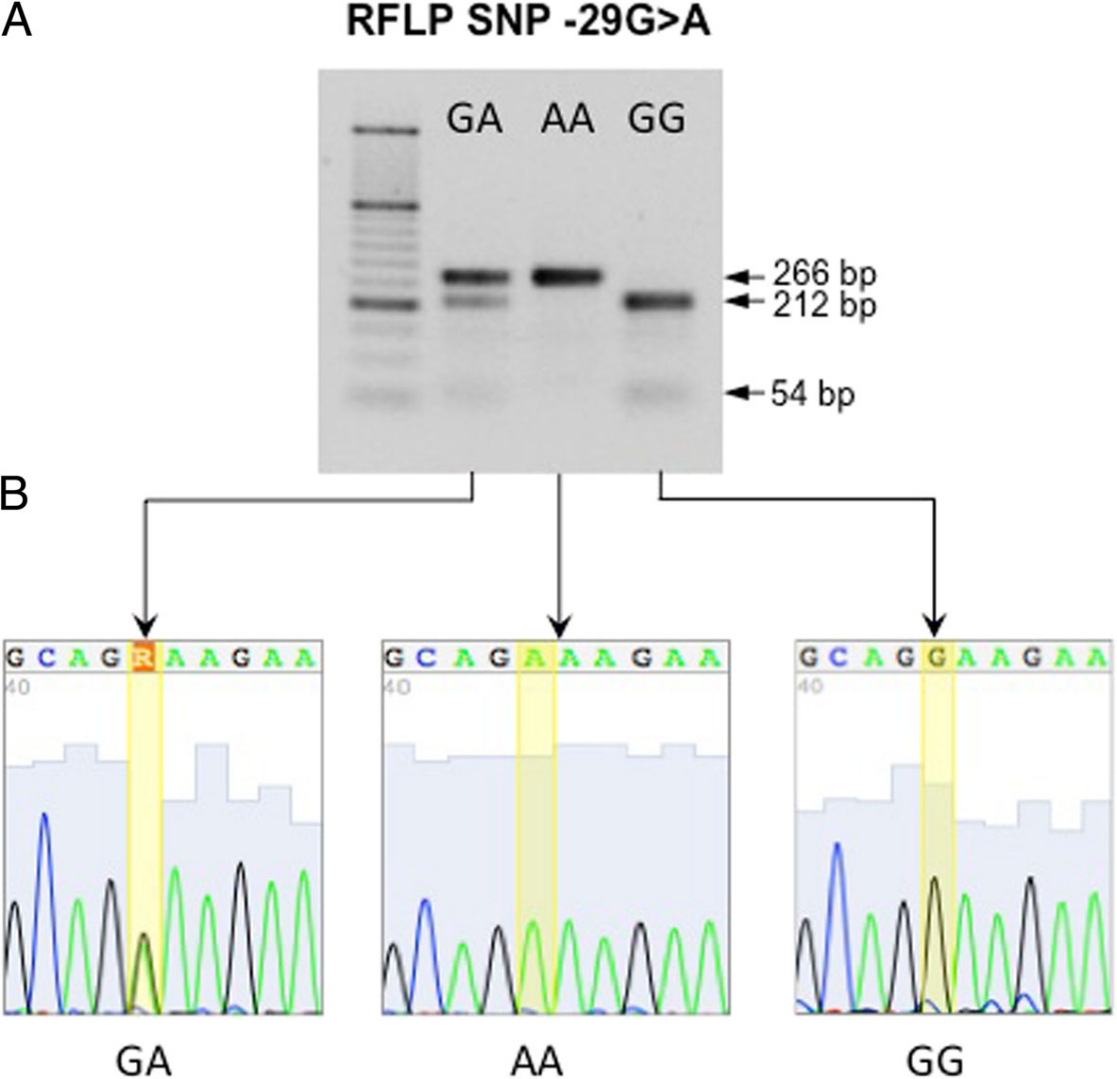
Restriction fragment length polymorphism (RFLP) analysis of the single nucleotide polymorphism at position − 29 of the *FSHR*. **a** Representative 3% agarose in TBE gel of the digested PCR products showing the migration of the bands corresponding to the GA, AA and GG genotypes. **b** Representative electropherograms obtained after DNA sequencing of the amplified PCR products bearing different genotypes at position − 29

The allelic and genotype frequency for the rs1394205 (−29G > A) SNP as assessed by RFLP (Fig. 2) are shown in Table 2. The frequencies of the G and A alleles and GG and AA genotypes in these groups were almost equally distributed (*p* = 0.309 and *p* = 0.296, for the allelic and genotype frequencies, respectively). Although the frequency of the GG genotype (22.5%) tended to be lower than that of the AA genotype (27.5%) in the donor group, and vice versa, the frequency of the latter genotype (20.1%) tended to be lower than that of the former (30.6%) in the group of infertile patients, the differences did not reach statistical significance (*p* = 0.296). Analysis for linkage disequilibrium of this SNP with those at positions c.919 (p.T307A) and c.2039 (p.N680S) in the total population studied, yielded low values of *D’* (0.360 and 0.477) and *r*^*2*^ (0.061 and 0.091) for both the rs6165-rs1394205 and rs6166-rs1394205 SNP pairs, respectively. Similar results were found when the donor and infertile patient groups were analyzed separately.

**Table 2:** Allele and genotype frequencies for the single nucleotide polymorphism (SNP) -29G > A of the *FSHR* in the population of normal oocyte donors and infertile Mexican mestizo women

Group	Allele frequency %	Genotype frequency %
Donors (*n* = 80)	G 47.5*	GG 22.5**
	A 52.5	GA 50.0
		AA 27.5
Infertile women (*n* = 144)	G 55.2	GG 30.6
	A 44.7	GA 49.3
		AA 20.1
TOTAL (*n* = 224)	G 52.4	GG 27.6
	A 47.5	GA 49.5
		AA 22.7

*P = 0.309 vs infertile women for the G and A alleles

**P = 0.296 vs infertile women for the GG, GA and AA genotypes

### Response to COS according to the N680S and -29G > A variants in the IVF group

Sixty-nine donors and 125 patients completed the COS cycle, and the data were analyzed to determine associations between the response to COS and particular *FSHR* SNP genotypes. All cases included in this secondary analysis exhibited complete linkage disequilibrium between the p.T307A and p.N680S variants. Tables 3 and 4 show the data on secondary outcomes in the oocyte donors and infertile patients grouped according to the SNPs at positions c.2039 and -29 at the *FSHR*, respectively*.* Normal donors conformed a quite homogenous group of women similar in age, and the COS protocol received was homogeneously distributed among the three SNP variants in positions c.2039 and -29 at the *FSHR*. Total FSH and LH administered, days of gonadotrophin administration until hCG injection, and serum E2 levels, did not differ significantly among normal women bearing the NN, NS or SS FSHR protein variants (Table 3) (*p* ≥ 0.122). Remarkably, the number of oocytes recovered from donors with the S680S variant were significantly lower than in those with the NS and NN genotypes (8.8 ± 1.3 vs. 13.9 ± 5.1 and 13.7 ± 5.1, respectively; *p* = 0.005), a difference that was maintained after Bonferroni correction (P_c_ = 0.001). In all cases, the S680S variant associated with lower number of oocytes retrieved corresponded to the combination AA/SS at positions 307/680 of the FSHR (see Fig. 1 for the distribution of this genotype combination).

**Table 3:** Total gonadotropin dose administered, response to COH in terms of serum estradiol levels and number of oocytes recovered, and stimulation days with gonadotropins until the day of hCG administration in oocyte donors and infertile women undergoing ART, according to the c.2039A **>** G (N680S) single nucleotide polymorphism at the *FSHR*. Numbers are means ± S.D., unless indicated

	Parameter	NN	NS	SS
*Oocyte donors* (*n* = 69)				
	Number	32	32	5
	Age	23.5* (18–29)	24 (20–29)	23 (21–25)
	Total FSH (IU)	1673.3 ± 658.2	1888.7 ± 702.4	1791.7 ± 286.7
	Total LH (IU)	1887.4 ± 917.4	1796.7 ± 826.4	1275.0 ± 742.5
	Estradiol (pg(ml)	2433.6 ± 1094.9	2607.6 ± 1315.9	2431.2 ± 501.8
	Oocyte number	13.7 ± 5.1	13.9 ± 5.14	8.8 ± 1.3^**^
	Stimulation days	9.9 ± 1.6	10.3 ± 1.6	10.6 ± 1.1
Infertile women (n = 125)				
	Number	54	57	14
	Age	35.5* (22–41)	36 (27–43)	34 (25–42)
	Total FSH (IU)	2329.7 ± 682.3	2261.2 ± 644.7	2203.4 ± 463.1
	Total LH (IU)	857.8 ± 639.9	700.7 ± 394.7	664.2 ± 350.3
	Estradiol (pg(ml)	2166.8 ± 1104.1	1977.6 ± 1028.0	2041.1 ± 970.6
	Oocyte number	9.2 ± 4.8	8.2 ± 5.7	9.8 ± 6.8
	Stimulation days	10.3 ± 1.4	10.2 ± 2.0	9.2 ± 1.8

*Median and range

^**^P = 0.005 vs NN and NS genotypes; the remaining parameters in the donors and all parameters in the infertile group did not differ significantly among genotypes (*P* ≥ 0.122 and *P* ≥ 0.135, for parameters in donors and infertile women, respectively)

**Table 4:** Total gonadotropin dose administered, response to COH in terms of serum estradiol levels and number of oocytes recovered, and stimulation days with gonadotropins until the day of hCG administration in oocyte donors and infertile women undergoing ART, according to the -29G > A single nucleotide polymorphism of the *FSHR*. Numbers are mean ± S.D., unless indicated

	Parameter	GG	GA	AA
Oocyte donors (*n* = 69)				
	Number	16	37	16
	Age*	24 (21–28)	24 (19–29)	23.5 (18–27)
	Total FSH (IU)	1874.3 ± 629.9	1681.3 ± 698.2	1921.6 ± 603.9**
	Total LH (IU)	1500.0 ± 698.7	1710.4 ± 905.8	2217.8 ± 797.1
	Estradiol (pg(ml)	2471.3 ± 1320.2	2445.6 ± 1069.2	2559.1 ± 1156.6
	Oocyte number	12.5 ± 4.5	13.9 ± 5.5	13.3 ± 4.5
	Stimulation days	10.3 ± 1.4	9.9 ± 1.7	10.5 ± 1.5
Infertile women (*n* = 125)				
	Number	40	59	26
	Age*	35 (27–42)	36 (22–43)	35.5 (26–39)
	Total FSH (IU)	2367.8 ± 655.95	2178.7 ± 599.05	2395.6 ± 696.4**
	Total LH (IU)	802.08 ± 637.9	708.1 ± 431.5	831.3 ± 478.9
	Estradiol (pg(ml)	2088.6 ± 1134.9	2116.93 ± 1039.6	1917.8 ± 966.9
	Oocyte number	8.8 ± 5.8	8.9 ± 5.4	8.7 ± 5.2
	Stimulation days	10.02 ± 1.7	10.0 ± 1.9	10.5 ± 1.5

*Median and range

**None of the parameters analyzed were statistically different among genotypes (*p* ≥ 0.711 and *p* ≥ 0.964 for parameters in the donor and infertile group, respectively)

In the infertile patients from the IVF group the causes of infertility included tubal factor (31.3% of total), endometriosis (9.0%), male factor (14.5%), PCOS with or without male factor (9%), age ≥ 39 years (17.4%), mixed (female/male) factor (0.7%), and unknown cause (18%). In this group of infertile patients, age, COS protocol received and diagnosis also were homogeneously distributed among the different genotypes at positions c.2039 and -29. Nevertheless, and in contrast with the normal group, no significant (*p* ≥ 0.135) differences in any of the secondary outcomes analyzed were detected among the three variants at position 680 of the FSHR.

None of the COS response parameters were significantly different among groups of donors (*p* = 0.711) and infertile patients (*p* = 0.964) carrying any of the three genotypes (GG, GA or AA) at position − 29 of the *FSHR* (Table 4).

### Frequency of N680S variants in Mayan women with low miscegenation

The frequencies of the A and G alleles of the 2039A > G SNP in this particular population were 65.5% and 34.5%, respectively. The genotype frequency of the minor GG variant was lower (7%) than that detected in the women from the IVF group (9.8%) and the Mexican mestizo population from SIGMA cohort (see below), albeit the difference did not reach statistical significance. Meanwhile, the genotype frequencies of the AA and AG variants in these Mayan women were 38% and 55%, respectively, not significantly different from those found in the other population groups studied (*p* = 0.792 for all three SNP genotypes). Using the surnames as markers for genetic testing, we found that the observed number of homozygous for the Mayan surnames (i.e. Mayan/Mayan paternal and maternal surnames) were lower than those expected by random mating (squared allelic frequencies of Mayan surname = 0.284 and 0.459, observed vs expected, respectively), confirming that the population studied for this particular SNP deviates from Hardy-Weinberg equilibrium as expected by their still preserved low miscegenation [41].

### Large database analysis

The large-scale genotype data set from 8182 Mexican mestizo subjects that fall on a cline of Native American and European Ancestry was additionally analyzed [35]. Since the frequency of the three *FSHR* SNPs analyzed was virtually the same between men and women and T2D and non-diabetic subjects, data from all subjects were considered together for the calculation of the allelic and genotype frequencies in these SNPs. The data confirmed the strong linkage disequilibrium between the c.919 and c.2039 SNPs in Mexican subjects (D’ = 0.91), and that both allelic and genotype frequencies (Table 5) were very similar to those detected in the IVF cohort, being the frequency of the corresponding GG genotypes 10.0% and 10.8%, respectively [vs 8.9% (*p* = 0.59); and 9.8% (*p* = 0.63) in the IVF population, respectively (see Table 1)]. For the -29G > A SNP, allelic and genotype frequencies were also very similar to those of the IVF cohort (Table 2), with a frequency of 25.6% for the AA genotype [vs 22.7% in the IVI population; (*p* = 0.38)] (Table 5). In subjects with more Native American ancestry (i.e. those falling within the quartile for the highest Native American ancestry as determined by PCA [36]) the frequency of the minor alleles at positions c.919 and c.2039, were lower by 15% and 9%, respectively, than in those with more European ancestry (c.919A > G = 23.0% and c.2039A > G = 28.0%, for subjects with more Native American ancestry vs c.919A > G = 38.0% and c.2039A > G = 37.0%, in subjects with more European ancestry; *p* < 0.001 for differences in both SNPs). Among a subgroup of 520 women in whom data of reproductive parameters was available, those carriers bearing the G allele at the c.2039 SNP tended to present lower pregnancy frequencies than women bearing the AA genotype, when stratified by either < 3 or ≥ 3 the total number of reported pregnancies per women [OR = 1.3, CI 95% 0.91–1.95 (*p* = 0.14)] (Additional file 1: Table S1)]. This cut-off value was based on data from the National Survey of Demographic Dynamics 2014 in Mexico (http://www.inegi.org.mx/proyectos/enchogares/especiales/enadid/2014/), in which the reported total fertility rate for 15- to 49-year-old Mexican women was 2.21. Further, this trend towards lower number of pregnancies was stronger after analyzing women older than 45 years (who comprised 92.5% of the total women population) separately by the proportion of Native American or European ancestry [OR = 2.0, C.I. 95% 1.03–3.90 (*p* = 0.04) for G allele carriers in the group with more Native American ancestry (*n* = 184 women); OR = 3.25, C.I. 95% 0.90–11.70 (*p* = 0.07) for carriers in the group with more European ancestry (*n* = 57 women] (Additional file 2: Tables S2 and Additional file 3: Table S3). When the models were adjusted for ethnicity, we observed that this factor was not statistically different (*p* > 0.05) between women with ≥3 pregnancies vs those reporting < 3 pregnancies, and thus ethnicity was not a confounder in the association between this outcome and genotypes (Additional file 4: Table S4).

**Table 5:** Allele and genotype frequencies for SNPs 307A > G, 680A > G, and -29G > A in an open Mexican mestizo population

SNP	Allele frequency %	Genotype frequency %
c.919A > G (p.T307A)	A (T) 69.2	AA (TT) 48.3
*n = 8207*	G (A) 30.8	AG (TA) 41.7
		GG (AA) 10.0
c.2039A > G (p.N680S)	A (N) 67.4	AA (NN) 45.6
*n = 8182*	G (S) 32.6	AG (NS) 43.6
		GG (SS) 10.8
-29G > A	G 50.4	GG 26.5
*n = 8195*	A 49.6	GA 47.9
		AA 25.6

There was no association between the number of pregnancies and the AA genotype at the -29G > A SNP nor between age at menarche or menopause and any of the *FSHR* SNPs analyzed in this large database.

## Discussion

In the present study, we determined the frequency of three FSHR/*FSHR* variants (p.T307A, p.N680S and -29G > A), in three groups of Mexican mestizo subjects. These populations, as well as those from other Latin American countries, are particularly unique in that their genetic structure contains an extensive, complex, and variable admixture between Africans, Native Americans, and Europeans (mainly Spaniards) that has significantly contributed to their corresponding phenotypic and genetic makeups [42, 43]. In women from the IVF cohort, we found a higher GG genotype frequency of the *FSHR* c.2039A > G SNP than those previously reported in placental samples from Mexican mestizo women (reported frequency, 5.9%) [23] as well as in the 1000 Genomes Project Phase 3 database [32](http://grch37.ensembl.org/) for a small cohort of Hispanic subjects residing in Los Angeles, CA, USA, of presumptive Mexican ancestry (frequency, 7.8%), but still markedly lower than in Caucasians, in whom the frequency range from ~ 20% to ~ 36% [6] (http://grch37.ensembl.org/). Further, in a population of fertile egg donors from Mediterranean origin residing in Spain, the frequency of this genotype is among the highest reported in Western Europe (42%) [15]. The frequency of the S680S FSHR variant observed in the present study (which was similar in normal oocyte donors and infertile patients), was also lower than that reported in Colombians (~ 14%)(http://grch37.ensembl.org/), in whom the estimated African and European genetic admixture proportions are higher than in Mexicans (11% vs. 5% and 60% vs. 37%, respectively) [42], thus emphasizing on the substantial impact of the admixture with Spaniards on the c.2039A > G *FSHR* SNP in Latin America. The even lower frequency of the GG genotype in Mayan women with low genetic admixture also points towards the genetic influence of Spaniards on the expression of this particular *FSHR* variant in Mexican mestizo women. If this assumption is correct and considering the similar frequency of the heterozygous (AG) genotype in the two populations studied (48% and 55% in the Mexican mestizo and Mayan women, respectively), then one might expect a progressive rise in GG genotype frequency as the admixure with non-Native American individuals increase in this particular Mayan population, which might confirm the Spaniard origin of this SNP in the Mexican mestizo population.

We additionally assessed the ovarian response to COS as well as the time and amount of gonadotrophins required to reach a mean follicle diameter of 18 mm in normal oocyte donors and infertile patients from the IVF group bearing different N680S FSHR variants. Despite the low number of oocyte donors with the GG genotype, we consistently detected an association of this genotype with a lower number of oocytes retrieved after gonadotrophin administration, thus confirming previous studies on the effect of the S680S phenotype on the ovarian sensitivity and response to exogenous FSH administration [6, 7, 15–17, 44–46]. The lower number of oocytes retrieved in donors with the GG genotype was not apparent in the infertile women, finding that may be due to the age-dependent vanishing effect of the N680S polymorphism on the ovarian response to COS, as previously suggested [6].

Another SNP that has been reported to influence the ovarian response to COS is the -29G > A polymorphism [4, 6, 7, 28]. In some studies the AA variant has been associated with reduced transcriptional activity of the *FSHR* and altered level of mRNA and receptor protein expression in vitro [47, 48] as well as with poor ovarian response to FSH during COS [4, 28, 47], although the latter has not been consistently found in other studies [31, 49]. The prevalence of the AA genotype varies depending on the geographic region considered, being relatively low in Caucasians [despite a relatively high frequency of the A allele in some European countries [31]], Africans, and Central-South Asians, and high in East Asians and Americans from both the USA and some Latin America countries [6] (http://grch37.ensembl.org/). In the present study, we found a relatively high prevalence of the AA genotype (~ 20% to 27%) in both donors and infertile patients of the IVF cohort, which was lower than that reported in the 1000 Genomes Project Phase 3 database for Mexican-American residents of the USA (~ 33%) (http://grch37.ensembl.org/). This difference in AA genotype frequency may be due to the relatively low number of samples genotyped and/or the particular genetic structure of the population included in that particular Project database. The frequency of the AA genotype detected in our normal oocyte donors also contrasts with those found in normo-ovulatory and infertile women from India (1% and 14%, respectively) [28], in whom the AA genotype was associated with poor ovarian response to COS as well as with primary and secondary amenorrhea [29]. More vividly, in the population of donors analyzed in the present study, those with the AA genotype did not show any significant difference in response to gonadotrophin administration compared with women exhibiting the GG or GA variants. Coexistence of and interactions with other ethnically-related SNPs at the *FSHR* or other genes involved in the ovarian response to gonadotrophins, may explain these apparent discrepancies among the various studies ([28, 29, 31], and present study).

Data extracted from a large database of SNPs in Mexican individual mestizo confirmed the allelic and genotype frequencies of the *FSHR* SNPs found in samples from the IVF group. Further, data on the number of pregnancies reported by carriers vs no carriers of the G allele at the c.2039A > G SNP, strongly suggests that the fertility potential of carriers of the G allele could be compromised. This might be due to the decreased sensitivity of the S680 FSHR variant to the gonadotrophic stimulus [10, 50] and the failure of the slight to moderate elevations in FSH levels to compensate for the abnormal function of the FSHR S680 variant, particularly in young women [9], as suggested by the longer menstrual cycle length exhibited by women homozygous for the G allele [13, 51]. The finding that the trend to decreased pregnancies in women bearing the G allele persisted even after stratification by the presence of more or less Native American/European ancestry, suggests that the effect of the Ser680 FSHR variant on reproductive potential results from the effect of Ser680 on FSHR function, rather than its interaction with other ethnically-related SNPs at the *FSHR* or other genes implicated in fertility. Overall, the results indicate that the frequency of the c.919A > G and c.2039A > G GG genotypes in Hispanic mestizo subjects are among the lowest reported [6] and remarkably similar to those found in large cohorts of Chinese women [16, 17], and that the presence of the Ser680 FSHR variant may impact on the reproductive potential of women when present in the homozygous state.

A major limitation of the present study is that the sample size in both IVF groups was not sufficiently powered to allow for detection of statistically significant differences in all secondary outcomes as it was not originally designed for this purpose. Nevertheless, we found that age, COS protocols and diagnosis were homogeneously distributed among all genotypes studied and that even after controlling for all these factors the significant difference on the number of oocytes retrieved from donors with the S680S FSHR persisted. Another drawback is that in the IVF groups, ethnicity index was not available and thus models were not adjusted for admixture. Nonetheless, using the UIDS cohort we found that ethnicity was not a confounder in the association between outcomes and genotypes, making valid these findings. Although this is the first large-scale analysis of the fertiliy potential in women with the *FSHR* SNPs analyzed, the information on reproductive events (mainly fertility potential as defined by the number of reported pregnancies) extracted from the large database of hispanic women also should be taken with caution as the questionaire applied was designed to obtain information on several metabolic aspects related to T2D, rather than on reproductive events and parameters that may influence, directly or indirectly, on the reproductive potential of the population studied. Thus, the data on the effect of the G allele at position c.2039 of the *FSHR* on fertility potential in the general population should be confirmed in other populations, particularly in those with a higher prevalence of this particular *FSHR* SNP variant than that reported herein. In this vein, Zilaitiene and colleagues [52] recently reported a significant association between the S680S FSHR variant and lower possibility of natural conception during the first 12 months of planned conception and other fertility parameters in a large population of young Caucasian women.

## Conclusions

The allele and genotype frequencies of the *FSHR* SNPs reported in this study add further information to the existing knowledge obtained from other genotyping projects. The frequency of the GG genotype at position c.2039 of the *FSHR* in Mexican mestizo subjects is among the lowest reported in the literature for both normal and infertile women. In oocyte donors receiving COS, expression of the S680S FSHR phenotype was associated with decreased number of oocytes recovered, whereas in women from the general population this SNP appears to influence on the fertility potential in carriers of the minor allele in terms of pregnancy rate. In contrast, the frequency of the AA genotype in position − 29 of the *FSHR* core promoter region in this particular population is among the highest reported and was not associated with significantly altered ovarian response to COS or particular reproductive events. Considering the absence of any deleterious effect of the − 29 AA genotype on the response to COS in Hispanic women, it is not advisable to perform this particular genotype testing to women from this population with the purpose of designing an individually-tailored protocol of gonadotrophin stimulation.

## Additional files

Additional file 1: **Table S1.** Number of pregnancies (according to < 3 or ≥ 3 pregnancies *per* women) in 52 Mexican mestizo women carriers of the AA genotype or G allele (AG plus GG genotypes) at the c.2039A > G SNP. (DOCX 15 kb)

Additional file 2: **Table S2.** Number of pregnancies (according to < 3 or ≥ 3 pregnancies *per* women) for each c.2039A > G SNP genotype in 184 Mexican mestizo women with more (4th quartile) Native American ancestry. (DOCX 15 kb)

Additional file 3: **Table S3.** Number of pregnancies (according to < 3 or ≥ 3 pregnancies *per* women) for each c.2039A > G SNP genotype in 57 Mexican mestizo women with more (4th quartile) European ancestry. (DOCX 15 kb)

Additional file 4: **Table S4.** ORs of being carrier of minor allele and having < 3 or ≥ 3 pregnancies (*n* = 520 Mexican mestizo women). Logistic regression models adjusted for age. (DOCX 14 kb)

## References

[1] Richards JS, Russell DL, Ochsner S, Hsieh M, Doyle KH, Falender AE (2002). Novel signaling pathways that control ovarian follicular development, ovulation, and luteinization. Recent Prog Horm Res.

[2] Huhtaniemi I (2015). A short evolutionary history of FSH-stimulated spermatogenesis. Hormones (Athens).

[3] Laan M, Grigorova M, Huhtaniemi IT (2012). Pharmacogenetics of follicle-stimulating hormone action. Curr Opin Endocrinol Diabetes Obes.

[4] Desai SS, Roy BS, Mahale SD (2013). Mutations and polymorphisms in FSH receptor: functional implications in human reproduction. Reproduction.

[5] Themmen APN (2005). An update of the pathophysiology of human gonadotrophin subunit and receptor gene mutations and polymorphisms. Reproduction.

[6] Simoni M, Casarini L (2014). Genetics of FSH action: a 2014-and-beyond view. Eur J Endocrinol.

[7] Casarini L, Santi D, Marino M (2015). Impact of gene polymorphisms of gonadotropins and their receptors on human reproductive success. Reproduction.

[8] La Marca A, Sighinolfi G, Argento C, Grisendi V, Casarini L, Volpe A (2013). Polymorphisms in gonadotropin and gonadotropin receptor genes as markers of ovarian reserve and response in in vitro fertilization. Fertil Steril.

[9] Perez Mayorga M, Gromoll J, Behre HM, Gassner C, Nieschlag E, Simoni M (2000). Ovarian response to follicle-stimulating hormone (FSH) stimulation depends on the FSH receptor genotype. J Clin Endocrinol Metab.

[10] Casarini L, Moriondo V, Marino M, Adversi F, Capodanno F, Grisolia C (2014). FSHR polymorphism p.N680S mediates different responses to FSH in vitro. Mol Cell Endocrinol.

[11] Tranchant T, Durand G, Piketty V, Gauthier C, Ulloa-Aguirre A, Crepieux P (2012). N680S SNP of the human FSH receptor impacts on basal FSH and estradiol level in women and modifies PKA nuclear translocation and CREB-dependent gene transcription in vitro. Hum Reprod.

[12] Simoni M, Tempfer CB, Destenaves B, Fauser BC (2008). Functional genetic polymorphisms and female reproductive disorders: part I: polycystic ovary syndrome and ovarian response. Hum Reprod Update.

[13] Greb RR, Grieshaber K, Gromoll J, Sonntag B, Nieschlag E, Kiesel L (2005). A common single nucleotide polymorphism in exon 10 of the human follicle stimulating hormone receptor is a major determinant of length and hormonal dynamics of the menstrual cycle. J Clin Endocrinol Metab.

[14] Behre HM, Greb RR, Mempel A, Sonntag B, Kiesel L, Kaltwasser P (2005). Significance of a common single nucleotide polymorphism in exon 10 of the follicle-stimulating hormone (FSH) receptor gene for the ovarian response to FSH: a pharmacogenetic approach to controlled ovarian hyperstimulation. Pharmacogenet Genomics.

[15] Lledo B, Guerrero J, Turienzo A, Ortiz JA, Morales R, Ten J (2013). Effect of follicle-stimulating hormone receptor N680S polymorphism on the efficacy of follicle-stimulating hormone stimulation on donor ovarian response. Pharmacogenet Genomics.

[16] Huang X, Li L, Hong L, Zhou W, Shi H, Zhang H (2015). The Ser680Asn polymorphism in the follicle-stimulating hormone receptor gene is associated with the ovarian response in controlled ovarian hyperstimulation. Clin Endocrinol (Oxf).

[17] Yan Y, Gong Z, Zhang L, Li Y, Li X, Zhu L (2013). Association of follicle-stimulating hormone receptor polymorphisms with ovarian response in Chinese women: a prospective clinical study. PLoS One.

[18] Laven JS, Mulders AG, Suryandari DA, Gromoll J, Nieschlag E, Fauser BC (2003). Follicle-stimulating hormone receptor polymorphisms in women with normogonadotropic anovulatory infertility. Fertil Steril.

[19] Anagnostou E, Mavrogianni D, Theofanakis C, Drakakis P, Bletsa R, Demirol A (2012). ESR1, ESR2 and FSH receptor gene polymorphisms in combination: a useful genetic tool for the prediction of poor responders. Curr Pharm Biotechnol.

[20] Genro VK, Matte U, De Conto E, Cunha-Filho JS, Fanchin R (2012). Frequent polymorphisms of FSH receptor do not influence antral follicle responsiveness to follicle-stimulating hormone administration as assessed by the follicular output RaTe (FORT). J Assist Reprod Genet.

[21] Klinkert ER, te Velde ER, Weima S, van Zandvoort PM, Hanssen RG, Nilsson PR (2006). FSH receptor genotype is associated with pregnancy but not with ovarian response in IVF. Reprod BioMed Online.

[22] Wang HS, Cheng BH, Wu HM, Yen CF, Liu CT, Chao A (2011). A mutant single nucleotide polymorphism of follicle-stimulating hormone receptor is associated with a lower risk of endometriosis. Fertil Steril.

[23] Dominguez-Lopez P, Diaz-Cueto L, Arechavaleta-Velasco M, Caldino-Soto F, Ulloa-Aguirre A, Arechavaleta-Velasco F (2018). The follicle-stimulating hormone receptor Asn680Ser polymorphism is associated with preterm birth in Hispanic women. J Matern Fetal Neonatal Med.

[24] Rendina D, Gianfrancesco F, De Filippo G, Merlotti D, Esposito T, Mingione A (2010). FSHR gene polymorphisms influence bone mineral density and bone turnover in postmenopausal women. Eur J Endocrinol.

[25] Qin X, Ma L, Yang S, Zhao J, Chen S, Xie Y (2014). The Asn680Ser polymorphism of the follicle stimulating hormone receptor gene and ovarian cancer risk: a meta-analysis. J Assist Reprod Genet.

[26] Corbo RM, Gambina G, Broggio E, Scacchi R (2011). Influence of variation in the follicle-stimulating hormone receptor gene (FSHR) and age at menopause on the development of Alzheimer's disease in women. Dement Geriatr Cogn Disord.

[27] Tuttelmann F, Laan M, Grigorova M, Punab M, Sober S, Gromoll J (2012). Combined effects of the variants FSHB -211G>T and FSHR 2039A>G on male reproductive parameters. J Clin Endocrinol Metab.

[28] Achrekar SK, Modi DN, Desai SK, Mangoli VS, Mangoli RV, Mahale SD (2009). Poor ovarian response to gonadotrophin stimulation is associated with FSH receptor polymorphism. Reprod BioMed Online.

[29] Achrekar SK, Modi DN, Meherji PK, Patel ZM, Mahale SD (2010). Follicle stimulating hormone receptor gene variants in women with primary and secondary amenorrhea. J Assist Reprod Genet.

[30] Simoni M, Nieschlag E, Gromoll J (2002). Isoforms and single nucleotide polymorphisms of the FSH receptor gene: implications for human reproduction. Hum Reprod Update.

[31] Wunsch A, Ahda Y, Banaz-Yasar F, Sonntag B, Nieschlag E, Simoni M (2005). Single-nucleotide polymorphisms in the promoter region influence the expression of the human follicle-stimulating hormone receptor. Fertil Steril.

[32] Auton A, Brooks LD, Durbin RM, Garrison EP, Kang HM, Korbel JO (2015). A global reference for human genetic variation. Nature.

[33] group. REA-SPcw (2004). Revised 2003 consensus on diagnostic criteria and long-term health risks related to polycystic ovary syndrome (PCOS). Hum Reprod.

[34] Canto P, Canto-Cetina T, Juarez-Velazquez R, Rosas-Vargas H, Rangel-Villalobos H, Canizales-Quinteros S (2008). Methylenetetrahydrofolate reductase C677T and glutathione S-transferase P1 A313G are associated with a reduced risk of preeclampsia in Maya-mestizo women. Hypertens Res.

[35] Williams AL, Jacobs SB, Moreno-Macias H, Huerta-Chagoya A, Churchhouse C, Marquez-Luna C (2014). Sequence variants in SLC16A11 are a common risk factor for type 2 diabetes in Mexico. Nature.

[36] Price AL, Patterson NJ, Plenge RM, Weinblatt ME, Shadick NA, Reich D (2006). Principal components analysis corrects for stratification in genome-wide association studies. Nat Genet.

[37] Sudo S, Kudo M, Wada S, Sato O, Hsueh AJ, Fujimoto S (2002). Genetic and functional analyses of polymorphisms in the human FSH receptor gene. Mol Hum Reprod.

[38] Brown H, Prescott R (2014). Generalised linear mixed models. Applied mixed models in medicine.

[39] Armstrong RA (2014). When to use the Bonferroni correction. Ophthalmic Physiol Opt.

[40] Barrett JC, Fry B, Maller J, Daly MJ (2005). Haploview: analysis and visualization of LD and haplotype maps. Bioinformatics.

[41] Lasker GW (1980). Surnames in the study of human biology. Am Anthropol.

[42] Ruiz-Linares A, Adhikari K, Acuna-Alonzo V, Quinto-Sanchez M, Jaramillo C, Arias W (2014). Admixture in Latin America: geographic structure, phenotypic diversity and self-perception of ancestry based on 7,342 individuals. PLoS Genet.

[43] Moreno-Estrada A, Gignoux CR, Fernandez-Lopez JC, Zakharia F, Sikora M, Contreras AV (2014). Human genetics. The genetics of Mexico recapitulates native American substructure and affects biomedical traits. Science.

[44] Tang H, Yan Y, Wang T, Zhang T, Shi W, Fan R (2015). Effect of follicle-stimulating hormone receptor Asn680Ser polymorphism on the outcomes of controlled ovarian hyperstimulation: an updated meta-analysis of 16 cohort studies. J Assist Reprod Genet.

[45] Casarini L, Pignatti E, Simoni M (2011). Effects of polymorphisms in gonadotropin and gonadotropin receptor genes on reproductive function. Rev Endocr Metab Disord.

[46] Alviggi C, Conforti A, Santi D, Esteves SC, Andersen CY, Humaidan P (2018). Clinical relevance of genetic variants of gonadotrophins and their receptors in controlled ovarian stimulation: a systematic review and meta-analysis. Hum Reprod Update.

[47] Desai SS, Achrekar SK, Pathak BR, Desai SK, Mangoli VS, Mangoli RV (2011). Follicle-stimulating hormone receptor polymorphism (G-29A) is associated with altered level of receptor expression in granulosa cells. J Clin Endocrinol Metab.

[48] Nakayama T, Kuroi N, Sano M, Tabara Y, Katsuya T, Ogihara T (2006). Mutation of the follicle-stimulating hormone receptor gene 5′-untranslated region associated with female hypertension. Hypertension.

[49] Tohlob D, Abo Hashem E, Ghareeb N, Ghanem M, Elfarahaty R, Byers H (2016). Association of a promoter polymorphism in FSHR with ovarian reserve and response to ovarian stimulation in women undergoing assisted reproductive treatment. Reprod BioMed Online.

[50] Nordhoff V, Sonntag B, von Tils D, Gotte M, Schuring AN, Gromoll J (2011). Effects of the FSH receptor gene polymorphism p.N680S on cAMP and steroid production in cultured primary human granulosa cells. Reprod Biomed Online.

[51] Gromoll J, Simoni M (2005). Genetic complexity of FSH receptor function. Trends Endocrinol Metab.

[52] Zilaitiene B, Dirzauskas M, Verkauskiene R, Ostrauskas R, Gromoll J, Nieschlag E (2018). The impact of FSH receptor polymorphism on time-to-pregnancy: a cross-sectional single-Centre study. BMC Pregnancy Childbirth.

