# Supplementary material for: Frequency of the T307A, N680S, and -29G>A single-nucleotide polymorphisms in the follicle-stimulating hormone receptor in Mexican subjects of Hispanic ancestry
Source: Reprod Biol Endocrinol. 2018 Oct 19;16:100. doi: 10.1186/s12958-018-0420-4 (PMC6195735; doi:10.1186/s12958-018-0420-4)
Supplement: Supplementary file 1 — Table S1. Number of pregnancies (according to < 3 or ≥ 3 pregnancies per women) in 52 Mexican mestizo women carriers of the AA genotype or G allele (AG plus GG genotypes) at the c.2039A > G SNP. (DOCX 15 kb) [file 12958_2018_420_MOESM1_ESM.docx]

| Genotype | Frequencies (%) | Deliveries | | TOTAL |
| --- | --- | --- | --- | --- |
|  |  | <3 | ≥3 |  |
| AA | *Within genotype*  *Between genotypes*  *Number of women* | 28.1  44.0  71 | 71.9  51  182 | 100%  253 (48.6%) |
| AG+GG | *Within genotype*  *Between genotypes*  *Number of women* | 34.5  56.0  92 | 65.5  49.0  175 | 100%  267 (51.4%) |
| TOTAL | *% total <3 and ≥*  *Between genotypes*  *Number of women* | 31.3  100%  163 | 68.7  100%  357 | 100%  520 (100%) |

Table S1. Number of pregnancies (according to <3 or ≥3 pregnancies *per* women) in 52 Mexican mestizo women carriers of the AA genotype or G allele (AG plus GG genotypes) at the c.2039A>G SNP.

OR 1.3 (0.92-1.94), p= 0.14
