# Supplementary material for: Frequency of the T307A, N680S, and -29G>A single-nucleotide polymorphisms in the follicle-stimulating hormone receptor in Mexican subjects of Hispanic ancestry
Source: Reprod Biol Endocrinol. 2018 Oct 19;16:100. doi: 10.1186/s12958-018-0420-4 (PMC6195735; doi:10.1186/s12958-018-0420-4)
Supplement: Supplementary file 2 — Table S2. Number of pregnancies (according to < 3 or ≥ 3 pregnancies per women) for each c.2039A > G SNP genotype in 184 Mexican mestizo women with more (4th quartile) Native American ancestry. (DOCX 15 kb) [file 12958_2018_420_MOESM2_ESM.docx]

Table S2. Number of pregnancies (according to <3 or ≥3 pregnancies *per* women) for each c.2039A>G SNP genotype in 184 Mexican mestizo women with more (4^th^ quartile) Native American ancestry.

| Genotype | Frequencies (%) | Pregnancies | | TOTAL |
| --- | --- | --- | --- | --- |
|  |  | <3 | ≥3 |  |
| AA | *Within genotype*  *Between genotypes*  *Number of women* | 20.9  34.6  18 | 79.1  51.5  68 | 100%  86 (46.7%) |
| AG and  GG* | *Within genotype*  *Between genotypes*  *Number of women* | 34.7  65.4  34 | 65.3  48.5  64 | 100%  98 (53.3%) |
| TOTAL | *% total <3 and ≥*  *Between genotypes*  *Number of women* | 28.3  100%  52 | 71.7  100%  132 | 100%  184 (100%) |
|  |  |  |  |  |

OR, 2.0 (1.03-3.90), p=0.04
