# Supplementary material for: Frequency of the T307A, N680S, and -29G>A single-nucleotide polymorphisms in the follicle-stimulating hormone receptor in Mexican subjects of Hispanic ancestry
Source: Reprod Biol Endocrinol. 2018 Oct 19;16:100. doi: 10.1186/s12958-018-0420-4 (PMC6195735; doi:10.1186/s12958-018-0420-4)
Supplement: Supplementary file 3 — Table S3. Number of pregnancies (according to < 3 or ≥ 3 pregnancies per women) for each c.2039A > G SNP genotype in 57 Mexican mestizo women with more (4th quartile) European ancestry. (DOCX 15 kb) [file 12958_2018_420_MOESM3_ESM.docx]

Table S3. Number of pregnancies (according to <3 or ≥3 pregnancies *per* women) for each c.2039A>G SNP genotype in 57 Mexican mestizo women with more (4^th^ quartile) European ancestry.

| Genotype | Frequencies (%) | Pregnancies | | TOTAL |
| --- | --- | --- | --- | --- |
|  |  | <3 | ≥3 |  |
| AA | *Within genotype*  *Between genotypes*  *Number of women* | 50.0  60.6  20 | 50.0  83.3  20 | 100%  40 (70.2%) |
| AG and  GG* | *Within genotype*  *Between genotypes*  *Number of women* | 76.5  39.4  13 | 23.5  16.7  4 | 100%  17 (29.8%) |
| TOTAL | *% total <3 and ≥*  *Between genotypes*  *Number of women* | 57.9  100%  33 | 42.1  100%  24 | 100%  57 (100%) |
|  |  |  |  |  |

OR, 3.25 (0.90-11.70 ), p=0.07
