# Supplementary material for: Frequency of the T307A, N680S, and -29G>A single-nucleotide polymorphisms in the follicle-stimulating hormone receptor in Mexican subjects of Hispanic ancestry
Source: Reprod Biol Endocrinol. 2018 Oct 19;16:100. doi: 10.1186/s12958-018-0420-4 (PMC6195735; doi:10.1186/s12958-018-0420-4)
Supplement: Supplementary file 4 — Table S4. ORs of being carrier of minor allele and having < 3 or ≥ 3 pregnancies (n = 520 Mexican mestizo women). Logistic regression models adjusted for age. (DOCX 14 kb) [file 12958_2018_420_MOESM4_ESM.docx]

Table S4. ORs of being carrier of minor allele and having <3 or ≥3 pregnancies (n= 520 Mexican mestizo women). Logistic regression models adjusted for age.

| SNP | Adjusted for ethnicity | | Without adjusting for ethnicity | |
| --- | --- | --- | --- | --- |
|  | OR | 95%CI | OR | 95%CI |
| rs6166 (C.2039A>G) | 1.33 | 0.91-1.95 | 1.35 | 0.92-1.97 |
| rs1374205  (-29G>A) | 1.05 | 0.67-1.65 | 0.99 | 0.64-1.54 |
